# Exogenous α-synuclein induces toll-like receptor 4 dependent inflammatory responses in astrocytes

**DOI:** 10.1186/s12868-015-0192-0

**Published:** 2015-09-07

**Authors:** Emmy H. Rannikko, Stephanie S. Weber, Philipp J. Kahle

**Affiliations:** Laboratory of Functional Neurogenetics, Department of Neurodegeneration, Faculty of Medicine, Hertie Institute for Clinical Brain Research, University of Tübingen, Otfried Müller Str. 27, 72076 Tübingen, Germany; German Center for Neurodegenerative Diseases, Tübingen, Germany; Division of Translational Alzheimer Neurobiology, Department of Neurobiology, Care Sciences and Society, Karolinska Institute, Stockholm, Sweden

**Keywords:** Synuclein, Toll-like receptor TLR4, Neuroinflammation, Cytokines, Nitric oxide synthase, Cyclooxygenase, MAP kinases, NF-κB, Endocytosis, Astrocytes

## Abstract

**Background:**

The pathological hallmarks of Parkinson’s disease are intracellular inclusions composed mainly of misfolded α-synuclein (αSYN). Under physiological conditions αSYN is mostly localized in synapses. In addition, a portion of αSYN is secreted to the extracellular space, where it may be sequestered by neighboring cells and could induce inflammatory responses. The mechanisms of αSYN internalization and signal transduction are not unequivocally clarified. In this work we investigated in primary mouse astrocytes the involvement of toll-like receptor 4 (TLR4) in the induction of inflammatory responses upon exposure to purified human αSYN produced in bacteria.

**Results:**

The mRNA induction of pro-inflammatory cytokines, inducible nitric oxide synthase and cyclooxygenase-2 was significantly reduced in TLR4 knockout astrocytes. The αSYN-mediated activation of c-Jun N-terminal kinases and p38 mitogen-activated protein kinase tended to be diminished, and nuclear translocation of the p65 subunit of nuclear factor κB was abolished in TLR4 knockout astrocytes. In contrast, the uptake of exogenous αSYN was unaffected by TLR4 knockout.

**Conclusions:**

Extracellular αSYN can activate pro-inflammatory TLR4 pathways in astrocytes, whereas αSYN uptake is independent of TLR4.

**Electronic supplementary material:**

The online version of this article (doi:10.1186/s12868-015-0192-0) contains supplementary material, which is available to authorized users.

## Background

Inflammation in the central nervous system is characterized by increased activation of microglia and astrocytes, elevated production of cytokines and other pro-inflammatory mediators including nitric oxide (NO) and prostaglandins, enhanced blood–brain-barrier permeability and hence increased leukocyte invasion. Neuroinflammation is an acutely protective mechanism, however, long-lasting and persistent formation and accumulation of pro-inflammatory mediators can initiate neuronal damage, neuronal circuit impairments and neurodegeneration. Correspondingly prolonged activation of neuroinflammation is thought to play a destructive role in many neurodegenerative diseases [1].

Parkinson’s disease (PD) is the most common neurodegenerative movement disorder, which affects approximately 1 % of the population over 65 years of age. Postmortem studies have shown an increase in neuroinflammatory signals in the brains of PD patients. Activated microglia and astrogliosis have been found in the affected substantia nigra of PD patients [2, 3]. Moreover, a higher density of CD8+ and CD4+ T-cells was shown in PD brains than in healthy control brains [4]. Correspondingly, the neuroinflammatory pathology found in brains of PD patients has been reproduced in several PD animal models [5].

Most of the glial cells in the brain are astrocytes, which are present in all regions of the brain and are localized in strategic positions in close proximity to neurons. Astrocytes are important contributors to the inflammatory responses during brain injury and infection. Similar to microglia, astrocytes can express and secrete a variety of molecules that modulate inflammation, for example toll-like receptors (TLRs), proinflammatory cytokines and NO. TLRs are receptors that are expressed in cells of the innate immune system. They recognize pathogen-associated molecular patterns and certain endogenous molecular patterns. The induction of inflammatory responses by the bacterial endotoxin lipopolysaccharide (LPS) is mediated by TLR4 [6]. Interestingly, TLR4 has been shown to be upregulated in microglia in synucleinopathy brains [7].

The pathological hallmarks of PD are intraneuronal protein inclusions called Lewy bodies or Lewy neurites. These inclusions mainly consist of the protein α-synuclein (αSYN) [8]. In addition, although astrocytes express only very low levels of αSYN themselves [9], αSYN-containing inclusions have been found in astrocytes in postmortem brains from patients with Lewy body diseases [10–12]. Moreover, point mutations and genomic multiplications of the gene encoding αSYN (*SNCA*) are linked to autosomal-dominant PD [13–15].

αSYN contains 140 amino acids and can associate with lipids [16–19]. The non-amyloid component domain (amino acids 65–95) of αSYN is essential for its pathogenic oligomerization and fibril formation [20]. αSYN is highly abundant in the brain, but is also present in other tissues, for example in red blood cells [21]. It is mainly localized in pre-synaptic terminals [19], and a small portion of the protein is also secreted to the extracellular space [22]. Cell stress like proteasomal dysfunction or oxidative stress may increase the secretion of αSYN [23, 24]. Extracellularly applied αSYN is able to induce inflammatory responses in neurons and glial cells [25]. Primary astrocytes treated with media from αSYN overexpressing SH-SY5Y neuroblastoma cells show increased expression of cytokines, among others the interleukins IL-6 and IL-1β, as well as cyclooxygenase-2 (COX-2) [26]. However, the molecular mechanisms by which αSYN induces neuroinflammatory responses are not entirely resolved. TLR4 was reported to mediate αSYN induction of tumor necrosis factor-α (TNF-α) and IL-6 in both microglia and astroglia [27]. On the other hand, TLR2 was reported to mediate microglial activation (proliferation and cytokine production) in response to cell-secreted αSYN [28], and β1-integrin signaling mediated the morphological and motility responses [29]. Moreover, pre-conditioning microglia with αSYN alters TLR responses [30].

The aim of this study was to investigate the role of TLR4 in the activation of inflammatory responses in astrocytes in the presence of extracellular αSYN. Primary astrocyte-rich cultures from TLR4 knockout mice and littermate controls were treated with purified recombinant human αSYN produced in bacteria. Moreover the internalization of extracellular αSYN into primary astrocytes was investigated. Our results indicate that αSYN is able to activate TLR4 signal transduction and thereby induces the expression of pro-inflammatory cytokines, inducible NO synthase (*Nos2*) and *Cox*-*2*, to mediate nuclear translocation of nuclear factor κB (NF-κB) and to activate c-Jun N-terminal kinase (JNK) and p38 mitogen-activated protein kinase (MAPK) modules. In contrast, the uptake of extracellular αSYN into astrocytes appears to be mediated by TLR4-independent pathway(s).

## Results

### Treatment with recombinant αSYN induces TLR4-dependent gene expression in primary astrocyte cultures

**Fig. 1:**
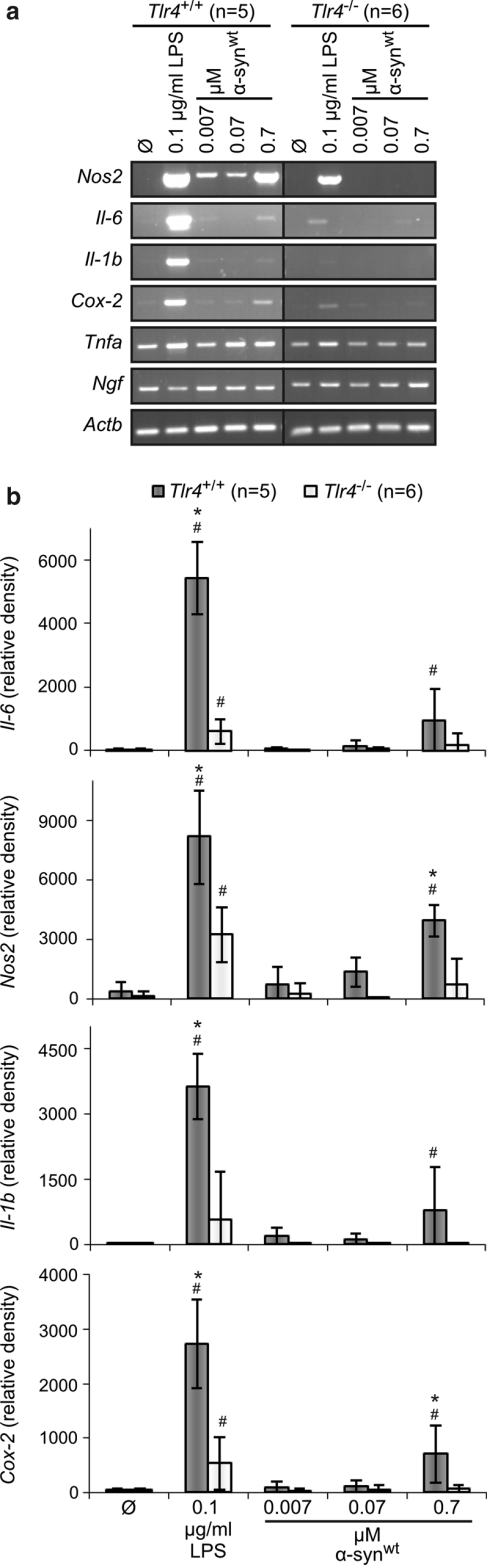
mRNA induction of pro-inflammatory mediators by extracellular αSYN is reduced in *Tlr4* ^−/−^ primary astrocytes. **a** Primary astrocyte-rich cultures from littermate *Tlr4* ^+/+^ and *Tlr4* ^−/−^ mice were treated for 72 h with indicated concentrations of LPS (as positive control) and αSYN, or left untreated (Ø). Total RNA was isolated from the cells and semi-quantitative PCR was performed with primers specific for the indicated gene products. Expression of β-actin mRNA (*Actb*) was measured as loading control. Images representative for 5–6 independent cultures are shown. **b** Ethidium bromide stained RT-PCR band signals were quantified with ImageJ software and normalized to *Actb*. *Error bars* indicate standard deviation, ^#^p < 0.05 compared to untreated samples (ANOVA Fisher’s PLSD), *p < 0.05 compared to *Tlr4* ^−/−^ (Student’s t test)

To investigate if TLR4 plays a role in inflammatory reactions to exogenous αSYN, primary littermate *Tlr4*^+/+^ and *Tlr4*^−/−^ astrocytes were treated with recombinant αSYN, and LPS as a positive control. The expression of *Nos2*, *Il*-*6*, *Il1b*, *Cox*-*2*, *Tnfa* and *Ngf* mRNA was investigated by semi-quantitative PCR. LPS dramatically induced the expression of *Nos2*, *Il*-*6*, *Il1b*, and *Cox*-*2* in *Tlr4*^+/+^ astrocytes (Fig. 1). *Tnfa* expression was also enhanced by LPS treatment while *Ngf* expression was less consistently altered. Though significantly reduced (Fig. 1b), some induction by LPS could be detected also in *Tlr4*^−/−^ astrocytes. This might reflect the ability of LPS to activate additional receptors, such as TLR2 [31]. Treatment with 0.7 µM αSYN induced the expression of *Nos2*, *Il*-*6*, *Il1b*, and *Cox*-*2* in *Tlr4*^+/+^ but no significant induction was detected in *Tlr4*^−/−^ astrocytes (Fig. 1b). *Tnfa* expression was elevated by αSYN in *Tlr4*^+/+^ astrocytes, but remained at basal levels in *Tlr4*^−/−^ astrocytes.

**Fig. 2:**
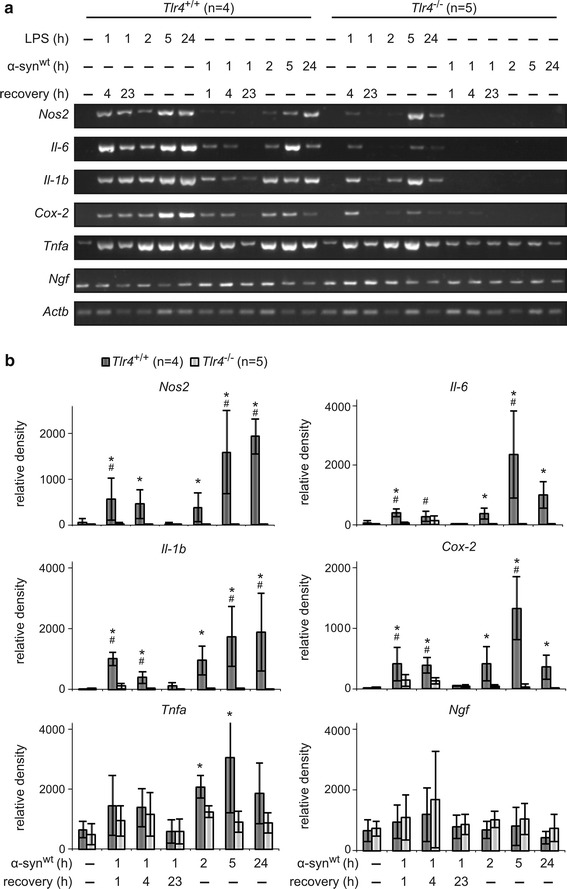
Time course and recovery of pro-inflammatory mediator expression. **a** Primary astrocyte-rich cultures from littermate *Tlr4* ^+/+^ and *Tlr4* ^−/−^ mice were treated for 1 h with 1.0 µg/ml LPS (as positive control) or 0.7 µM αSYN, after which the cells were allowed to recover in growth media for 1, 4 or 23 h. Parallel cultures were continuously treated with 1.0 µg/ml LPS or 0.7 µM αSYN for 2, 5 or 24 h. Control cells were left untreated (-). Total RNA was isolated and semi-quantitative PCR was performed as in Fig. 1a. Images representative for 4–5 independent cultures are shown. **b** RT-PCR band quantifications were done as described for Fig. 1b. *Error bars* indicate standard deviation, ^#^p < 0.05 compared to untreated samples (ANOVA Fisher’s PLSD), *p < 0.05 compared to *Tlr4* ^−/−^ (Student’s t test)

Time course experiments confirmed the inductions within few hours of *Nos2* and *Cox*-*2* as well as *Il*-*6* and *Il1b*, which were abolished in *Tlr4*^−/−^ astrocytes (Fig. 2). *Tnfa* was similarly induced by αSYN while again only basal expression remained in *Tlr4*^−/−^ astrocytes. Washout of agonists showed the reversible nature of astrocyte stimulations (Fig. 2). We also measured NO release from stimulated astrocytes (Additional file 1: Figure S1). Similar to LPS, αSYN treatment clearly induced NO release, although overall the variance of this assay was high. Nevertheless, administration of viper peptide that inhibits TLR4 largely abolished NO release, in contrast to the control peptide (Additional file 1: Figure S1). In conclusion, the mRNA induction of pro-inflammatory mediators by extracellularly applied αSYN involves TLR4.

### Decreased p38 and JNK phosphorylation in *Tlr4*^−/−^ astrocytes after αSYN treatment

Since mRNA inductions of inflammatory mediators by extracellular αSYN appeared to depend on the presence of TLR4 we wondered if other pathways that are activated by TLR4 were also affected. Therefore we treated primary astrocytes from littermate *Tlr4*^+/+^ and *Tlr4*^−/−^ mice with LPS as positive control or recombinant human αSYN for 1 h. The cells were recovered in growth media for 0–24 h (Additional file 2: Figure S2A) and immunoblotting was performed using antibodies against phosphorylated (activated) p38 MAPK and JNK. In *Tlr4*^+/+^ cells treatment with αSYN led to phosphorylation of p38 MAPK and JNK whereas in *Tlr4*^−/−^ less induction of the phosphorylation was seen. In another experiment the astrocytes were continuously treated with LPS and αSYN for 0–24 h (Additional file 2: Figure S2B). Also in this experiment we could detect increased phosphorylation of p38 MAPK and JNK after 2–6 h in *Tlr4*^+/+^ astrocytes. Again the induction of p38 MAPK and JNK phosphorylation tended to be reduced in *Tlr4*^−/−^ astrocytes. In conclusion, treatment of astrocyte-rich primary cell cultures with recombinant αSYN induced phosphorylation of p38 MAPK and JNK, involving TLR4.

### Extracellularly applied αSYN does not cause to nuclear translocation of NF-κB in *Tlr4*^−/−^ astrocytes

**Fig. 3:**
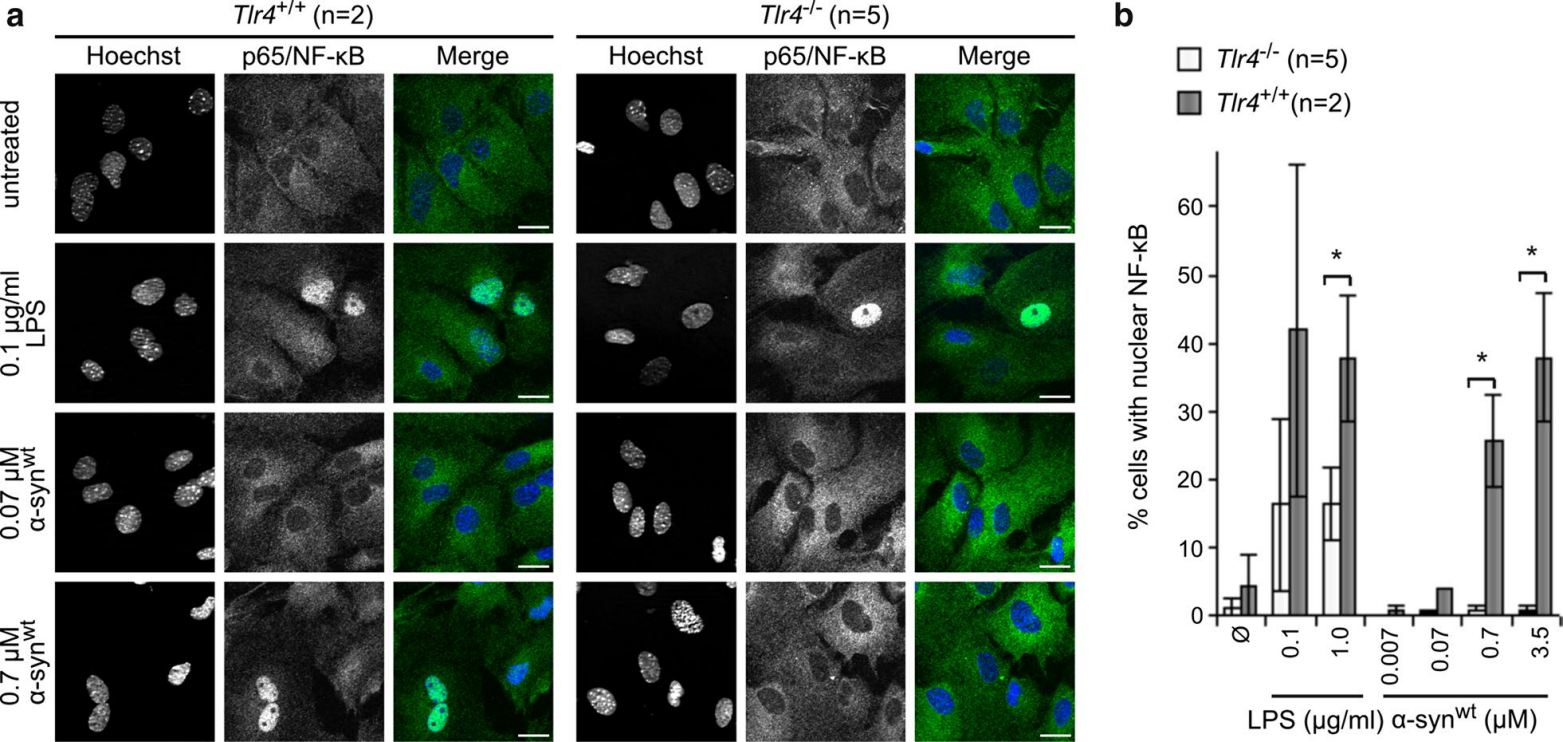
αSYN treatment induces TLR4 dependent nuclear translocation of NF-κB in primary astrocytes. **a** Primary astrocyte-rich cultures from littermate *Tlr4* ^+/+^ and *Tlr4* ^−/−^ mice were treated with indicated concentrations of LPS (as positive control) and recombinant human αSYN for 6 h or left untreated. The cells were fixed and immunostained using an antibody specific for p65 (*green*). Nuclei were counterstained with Hoechst 33342 (*blue*). Images were obtained with 25× objective using Axioimager microscope equipped with ApoTome Imaging system (Carl Zeiss). *Scale bars* correspond to 20 µm. **b** p65/NF-κB positive versus total cells were quantified from images as shown in (**a**). 200–400 cells per sample were analyzed. *Error bars* show standard deviation, *p < 0.05 (Student’s t test),* n* is indicated in the figure

Activation of TLR4 leads to phosphorylation and degradation of IκB and thus allows the transcription factor complex NF-κB to translocate to the nucleus. Primary *Tlr4*^+/+^ and *Tlr4*^−/−^ astrocytes were treated with different concentrations of the positive control LPS or recombinant human αSYN for 6 h. The cells were immunostained for p65, a component of the class II NF-κB protein complex, and p65 positive nuclei were quantified. As expected, LPS treated cells showed an increased number of p65 positive nuclei in both *Tlr*4^+/+^ and *Tlr4*^−/−^ cells, however to a lesser extent in the knock out astrocytes (Fig. 3). The recombinant αSYN induced NF-κB translocation in *Tlr4*^+/+^ cells in a concentration dependent manner, whereas in *Tlr4*^−/−^ astrocytes very few p65 positive nuclei were detected. Thus indicating that extracellular αSYN can induce the nuclear translocation of NF-κB in a TLR4-dependent manner.

### Primary astrocytes internalize αSYN from the medium in a TLR4-independent manner

**Fig. 4:**
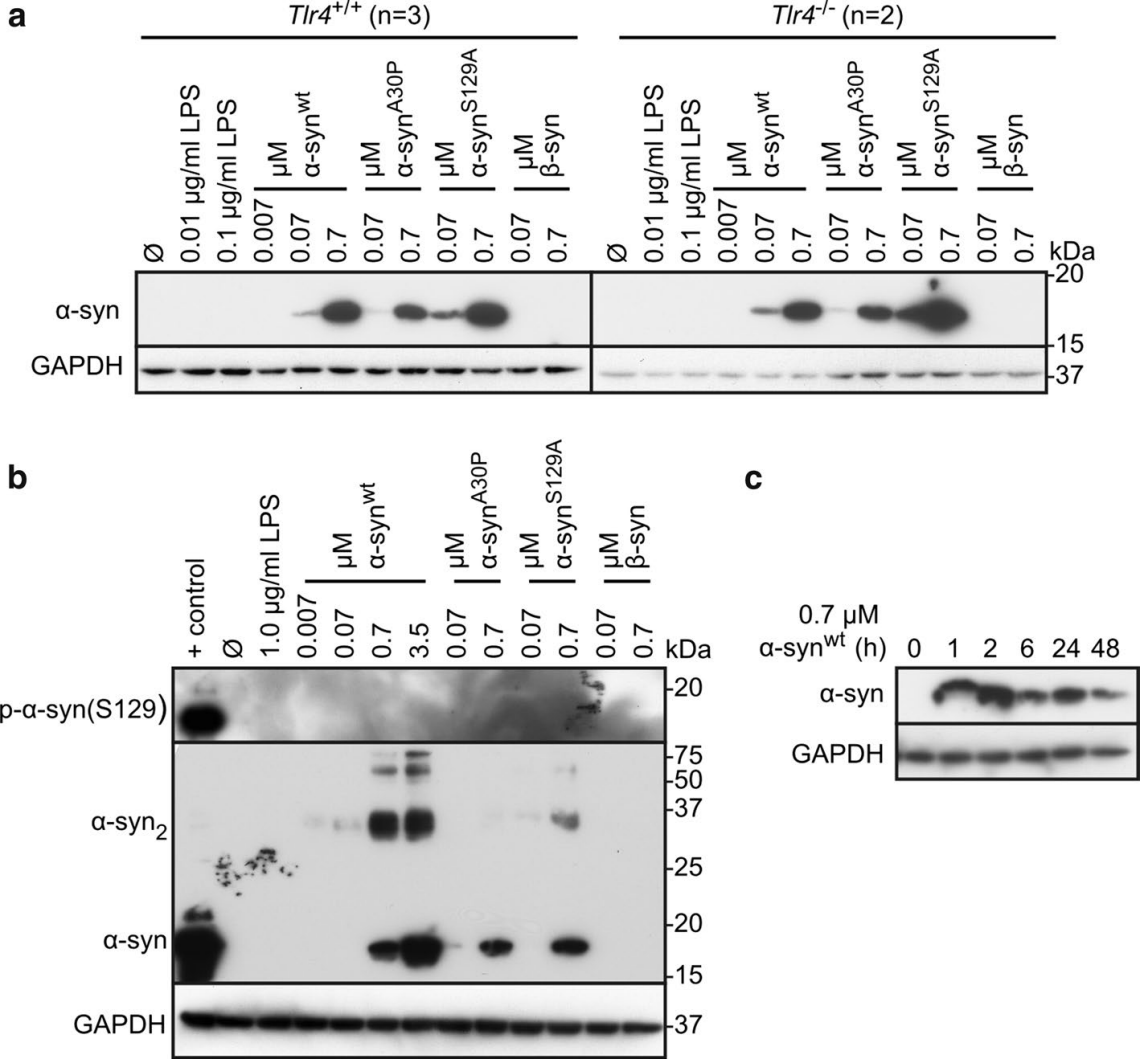
Extracellular αSYN is internalized by astrocytes in a TLR4 independent manner. Primary astrocytes from littermate *Tlr4* ^+/+^ and *Tlr4* ^−/−^ mice (**a**) or *Tlr4* ^+/+^ mice (**b**) were continuously treated for 48 h with indicated concentrations of recombinant human synucleins or LPS or left untreated (Ø). Cells were washed twice with PBS prior to lysis. The protein lysates were immunoblotted and probed with human-specific anti-αSYN (**a**, **b**) and also antibody against αSYN phosphorylated at S129 (**b**). GAPDH serves as control for equal protein loading throughout. Positive control in (**b**) is brain lysate from a 18 month old Thy1[A30P]hSNCA transgenic mouse, which has high amounts of phosphorylated αSYN. N is indicated in the figure (**a**) or N = 8 (**b**, **c**). **c** Primary wild type astrocytes were treated continuously with 0.7 µM αSYN for indicated times. Protein lysates were examined by immunoblotting as above

Different studies have indicated that a variety of cell types, for example neuronal cells, microglia and astrocytes, are able to internalize extracellular αSYN [26, 32]. The mechanism of the uptake of the monomeric form has been suggested to differ from the uptake of oligomeric and fibrillar αSYN [33]. We investigated if primary *Tlr4*^+/+^ and *Tlr4*^−/−^ astrocytes cultures were able to internalize the recombinant αSYN. Indeed, after 48 h of continuous incubation an immunoblot signal for human αSYN could be detected in samples that had been treated with 0.07 or 0.7 µM wild-type αSYN^wt^, mutant αSYN^A30P^ or phosphorylation-deficient αSYN^S129A^ (Fig. 4a). No considerable difference in the internalization of different αSYN variants or between the genotypes of astrocytes could be detected. Pathological αSYN is extensively phosphorylated at S129 and at this site phosphorylated αSYN is found in Lewy bodies [34]. To see if the internalized αSYN is phosphorylated in the astrocytes we treated primary astrocyte cultures with recombinant αSYN for 48 h. No signal could be detected with a phospho-S129 αSYN specific antibody (Fig. 4b). As a positive control for the antibody we used brain lysate from an aged *SNCA* transgenic mouse, which harbors excessive amounts of S129 phosphorylated αSYN [35]. Also the αSYN^S129A^ mutant was internalized (Fig. 4b), suggesting that phosphorylation at S129 is not a predominant factor in astrocytic uptake of αSYN.

Next we made a time course experiment where primary astrocyte cultures were treated continuously for 0–48 h with recombinant human αSYN. Already after 1 h the astrocytes had internalized detectable amounts of the recombinant αSYN. Interestingly, the intensity of the signal from internalized αSYN diminished with time (Fig. 4c). Thus, astrocytes seem to be able to purge extracellular αSYN.

## Discussion

The molecular mechanisms that trigger degeneration of the dopaminergic neurons in PD are still under investigation. It is currently heavily debated if αSYN can act as an agent that spreads neurodegeneration in PD brains and thus cause the progression of the disease. The role of the portion of αSYN that is secreted from neurons is largely unknown. Previous studies have shown that treatment with recombinant αSYN produced in bacteria as well as cell culture medium from cells that overexpress αSYN induces neuroinflammatory responses in microglia and astroglia [26, 27, 36–38]. In agreement with this we could see an induction of cytokines, *Cox*-*2* and *Nos2* mRNA, phosphorylation of p38 MAPK and JNK, and nuclear translocation of NF-κB in primary astrocyte rich cultures that had been treated with recombinant human αSYN purified from bacteria. Previous studies have suggested that TLR4 may be involved in synucleinopathies. Upregulation of TLR4 has been detected in multiple system atrophy, both in the brains of patients with the disease and in brains from a transgenic mouse model of the disease [7, 39]. Specifically, Fellner et al. [27] recently showed that αSYN preparations greatly enhanced rapid secretion of TNF-α and C-X-C motif chemokine ligand 1, and a more delayed secretion of IL-6 in *Tlr4*^+/+^ astrocytes, which was strongly suppressed in *Tlr4*^−/−^ astrocytes. Our present study confirms these findings at the mRNA level for the delayed Il-6 response, and adds *Il1b* to the list of TLR4-dependent αSYN responses. Moreover, we identify *Nos2* and *Cox*-*2* as potential signaling targets, and show the activation of MAPK and NF-κB pathways by which TLR4 appears to mediate pro-inflammatory responses to extracellular αSYN in astrocytes.

Astrocytes are in close proximity to neurons and neuronal synapses. These cells have been shown to be important scavengers of different potentially neurotoxic molecules that are secreted from neurons like glutamate, potassium and calcium. In accordance with this, we could detect a fast astrocytic internalization of the applied extracellular αSYN followed by degradation. These findings are supported by a previous study where cell secreted αSYN was readily endocytosed by primary rat astrocytes [26]. This implicates that astrocytes are decreasing the amount of potentially proinflammatory extracellular αSYN. The exact mechanism of the uptake of αSYN is still being investigated. In addition, the mechanism of uptake of monomeric and protofibrillar αSYN has been suggested to differ [33]. Monomeric αSYN has been suggested to be able to passively diffuse through the plasma membrane [40]. Thus αSYN would be localized in the cytosol and be directly available for proteasomes. However, also lipid-raft mediated endocytosis has been suggested for the uptake of monomeric αSYN [41]. The reproducible TLR4-independent αSYN uptake route in astroglial cells observed here and by Fellner et al. [27] remains to be further characterized. It is presently unknown if other receptor(s) mediate αSYN endocytosis in astrocytes, or if it is a receptor-independent form of pinocytosis, or a non-classical internalization or membrane penetration mechanism.

The formation and biological activities of oligomeric and fibrillar αSYN species are a vast topic in the field. Specifically, pre-formed fibrils are suggested to propagate cell-to-cell spread of αSYN [42, 43], and specific types of αSYN oligomers were reported to activate TLR2 on microglia [28]. On the other hand, “soluble” αSYN was a powerful stimulator of TLR4 both on microglia and astrocytes [27]. Cell type and receptor specificities, experimental conditions or conceptual issues could account for such differences. The present study did not focus on the nature of αSYN species involved in TLR4 responses and uptake in astrocytes. No procedures were undertaken to pre-aggregate our αSYN preparations, but we cannot rule out the presence of some αSYN oligomeric or aggregated species. Moreover, it is hard to predict what happens on the membrane surface, and if αSYN oligomers assemble near or on TLR4, or if αSYN oligomerizes during or after uptake. More work is necessary to unravel the different roles of the various αSYN assemblies in the brain.

The αSYN level has been measured to be 0.01 or 0.035 nM in the interstitial fluid of control or transgenic *SNCA* mice, respectively [44]. The inflammatory responses were seen when we used 0.7 µM of αSYN, which is a more than 20,000 times higher concentration. However, it is possible that the local concentration within synapses, which are touched by astrocytic endfeet, can be at least temporarily increased. It would be very interesting to be capable of measuring the αSYN concentration within synapses and to investigate the effect of physiologically relevant extracellular αSYN concentrations.

## Conclusions

Extracellular αSYN can induce inflammatory responses in astrocytes in a TLR4-dependent manner and this may worsen the stress conditions in synucleinopathy brains. On the other hand, astrocytes may also play a protective role in the disease progression by efficiently sequestering and degrading the proinflammatory extracellular αSYN.

## Methods

### Antibodies

Primary antibodies used were: rat monoclonal against human αSYN (15G7 [45]; provided by Dr. Elisabeth Kremmer, Helmholtz Center, Munich, Germany), mouse monoclonal anti-βSYN (Syn207; Millipore), mouse monoclonal against glyceraldehyde-3-phosphate dehydrogenase (GAPDH) (6C5; Meridian Life Sciences), rabbit monoclonal 3D7 against phospho-p38 MAPK (T180/Y182), rabbit polyclonal anti-p38 MAPK, rabbit polyclonal against phospho-JNK (T183/Y185), rabbit monoclonal 56G8 anti-JNK (all from Cell Signaling Technology), F-6 mouse monoclonal against NF-κB p65 (Santa Cruz Biotechnology) and rabbit monoclonal EP1536Y against αSYN phosphorylated at S129 (Abcam). Secondary peroxidase conjugated antibodies were purchased from Jackson ImmunoResearch Laboratories and secondary Alexa-Fluor conjugated antibodies were purchased from Invitrogen GmbH.

### Synuclein expression constructs

pET30a *SNCB* was a kind gift from Hilal Lashuel at the Swiss federal institute of technology in Lausanne, Switzerland. To create bacterial expression vectors *SNCA* wt, A30P and S129A were cloned from pCDNA3.1-SNCA [46] into *Nhe*I/*Hind*III site of pET21a vector (Novagen). The nucleic acid sequences of the constructs were confirmed by sequencing using BigDye Terminator v3.1 according to the manufacturer’s instructions. Reaction products were analyzed using an ABI 3100 Genetic Analyzer.

### Preparation of recombinant αSYN

DYT medium (1.6 % (w/v) Tryptone, 1 % (w/v) Yeast extract, 0.5 % (w/v) NaCl) supplied with 100 µg/ml ampicillin was inoculated with *Escherichia Coli* BL21 Rosetta2 (Stratagene) transformed with pET21a-SNCA wt or mutants or pET30a-SCNB. The bacteria were grown at 37 °C at 200 rpm to an optical density between 0.5 and 0.8 at 590 nm. Protein expression was induced by 0.5 mM isopropyl-β-d-thiogalactopyranoside for 4–6 h at 37 °C at 200 rpm. The bacteria were centrifuged at 3500×*g* for 10 min at 4 °C and the cell pellet was stored at −20 °C.

The bacterial pellet was gently diluted in a 10 mM phosphate buffer (pH 7.4) containing 25 mM NaCl and EDTA-free Cømplete protease inhibitor (Roche Diagnostics). The cells were homogenized in a French press (EmulsiFlex-C5; AVESTIN) and sonicated three times for 30 s at 50 % input (SONOPULS HD 2070; BANDELIN). The solutions were incubated for 15 min at 95 °C. The denatured proteins were removed by two time centrifugation at 17,000×*g* at 4 °C for 30 min. The supernatant was applied on a Q-Sepharose column (50 ml, diameter 23 mm) and eluted with a two column volumes long 25 mM to 1000 mM NaCl gradient in 10 mM phosphate buffer (pH 7.4). Fractions with strong synuclein signals on dot blots were desalted on a Sephadex S-200 column (column volume 180 ml, diameter 16 mm) using a 10 mM phosphate buffer containing 150 mM NaCl, (pH 7.4). The synuclein content in the eluted fractions was monitored on dot blots. Absence of contaminating proteins was examined in 2–5 µl samples by a sensitive variant of Coomassie staining (10 % (v/v) ethanol, 30 mM HCl and 0.3 mM Coomassie Brilliant Blue G250). Fractions that were positive for an immunoblot synuclein signal and had only one visible protein band in the Coomassie gel were pooled, aliquoted and stored at −20 °C. The absorbance at 280 nm was measured and the synuclein concentrations were calculated using the extinction factors ε(280, 0.1 %) 0.354 for αSYN and 0.417 for βSYN (calculated with the ProtParam tool of ExPASy, Swiss Institute of Bioinformatics). Characterization of recombinant synucleins is shown in Additional file 3: Figure S3.

Endotoxin levels of the recombinant proteins were estimated using PYROGENT Plus Single Tests (sensitivity 0.06 EU/ml) following the manufacturer’s instructions (LONZA). Briefly, Limulus Amebocyte Lysate was dissolved in 250 μl of diluted protein samples (1:1, 1:10 and 1:25), or endotoxin-free water, then incubated for 60 min at 37 °C.

### Mouse maintenance and preparation of primary astrocytes

Mice were kept under standard conditions with free access to food and water in a cycle of 12 h of light and 12 h of dark. The local animal welfare committee, Referat 35, *Regierungspräsidium* Tübingen approved all experiments and procedures. Number of animals used and their suffering was kept to a minimum.

Heterozygous *Tlr4*^+/−^ mice [47] were intercrossed. Astrocyte-rich primary cultures were prepared as described previously [48]. Newborn pups were decapitated, and whole brains were removed. The brain from each pup was handled separately. Cells were separated in preparation buffer (137 mM NaCl, 5.4 mM KCl, 0.2 mM KH_2_PO_4_, 0.2 mM Na_2_HPO_4_, 1 g/l glucose, 20 g/l sucrose, 50 µg/ml gentamycin) by mechanically forcing the brains through nets with a mesh width of first 250 µM and second 135 µm. The cells were resuspended in Dulbecco’s modified Eagle’s medium (Biochrom) supplemented with 10 % fetal calf serum, 10 U/ml penicillin G and 10 µg/ml streptomycin sulfate. Cells were cultured in 75 cm^2^ cell culture bottles at 37 °C in 5 % CO_2_ in a humidified atmosphere. Each individual culture was genotyped by PCR of genomic DNA (primers *Tlr4*^+/+^rev 5′-CGTGTAAACCAGCCAGGTTTTGAAGGC-3′, *Tlr4*^+/+^for 5′-TGTTGCCCTTCAGTCACAGAGACTCTG-3′, *Tlr4*^−/−^rev 5′-TGTTGGGTCGTTTGTTCGGATCCGTCG-3′). After 1 week in culture the flasks containing the astrocytes were shaken at 37 °C at 180 rpm over night. Then the cells were washed once with phosphate buffered saline (PBS, pH 7.4; 2.2 mM KH_2_PO_4_, 7.8 mM Na_2_HPO_4_, 150 mM NaCl) and fresh growth medium was supplied. After one additional week the cells were plated in appropriate cell culture dishes. In order to avoid stress the astrocytes were allowed to normalize for 4–7 days and they were supplied with fresh media 1 or 2 days before use in experiments. The cells were incubated in growth media unless stated otherwise supplemented with indicated concentrations of LPS or recombinant synuclein for indicated times.

### Semi-quantitative PCR

Cells were lysed in RLT-RNA lysis buffer at −20 °C. RNA was isolated using the RNeasy Mini kit (Qiagen). cDNA was produced with Transcriptor High Fidelity cDNA Synthesis kit (Roche Diagnostics) and anchored-oligo(dT)_18_ primer. The cDNA was used as template for PCR reactions with given primers (*Actb*: 5′-CTAAGGCCAACCGTGAA-3′ and 5′-CCGGAGTCCATCACAAT-3′, *Il1b*: 5′-CAGGCAGGCAGTATCACTCA-3 and 5′-AGGCCACAGGTATTTTGTCG-3′, *Il*-*6*: 5′-GTTCTCTGGGAAATCGTGGA-3 and 5′-GGAAATTGGGGTAGGAAGGA-3′, *Cox*-*2*: 5′-TCCTCCTGGAACATGGACTC-3′ and 5′-CCCCAAAGATAGCATCTGGA-3′, *Tnfa*: 5′-AGCCCCCAGTCTGTATCCTT-3′ and 5′-AGCAAAAGAGGAGGCAACAA-3′, *Ngf*: 5′-GCAGTGAGGTGCATAGCGTA-3′ and 5′-CACTGAGAACTCCCCCATGT-3′, *Nos2*: 5′-GTGGTGACAAGCACATTTGG-3′ and 5′-GGCTGGACTTTTCACTCTGC-3′). The DNA fragments were separated in 1.5 % (w/v) agarose gels containing ethidium bromide. DNA bands were detected with a Vilber Lourmat. Signals were quantified by densitometry using ImageJ software.

### Western immunoblot analysis

Cells were lysed for 30 min on ice in lysis buffer (1 % Triton X-100, 50 mM Tris–HCl (pH 7.6), 150 mM sodium chloride, 10 mM sodium pyrophosphate, 2 mM EDTA and Cømplete protease inhibitor cocktail from Roche Diagnostics). Cell debris was removed by centrifugation (15 min at 14,000×*g* and 4 °C) and protein concentrations of lysates were determined using a bicinchoninic acid protein assay kit (Pierce). Samples were diluted in Laemmli buffer, boiled at 95 °C for 5 min, and centrifuged for 20 s at 16,100×*g* before loading. A total of 10–25 µg proteins were then subjected to denaturing 10 or 15 % polyacrylamide gel electrophoresis and transferred onto Immobilon-P polyvinylidene difluoride membranes (EMD Millipore). Membranes were pre-incubated for 1 h at 20 °C in 5 % skim milk in TBS-T [50 mM Tris–HCl (pH 7.4), 150 mM NaCl and 0.1 % Tween-20]. Primary antibodies diluted in Western Blocking Reagent (Roche Diagnostics) were incubated over night at 4 °C. Then membranes were washed 3–5 times in TBS-T. Horseradish peroxidase-conjugated secondary antibodies in TBS-T supplemented with 5 % milk were incubated for 1 h at 20 °C and membranes washed again 3–5 times with TBS-T. Detection of proteins was performed with the Immobilon Western Chemiluminescent HRP Substrate (EMD Millipore) on Amersham Hyperfilm™ for enhanced chemiluminescence (GE Healthcare).

### NF-κB nuclear translocation

Primary astrocytes were cultured on poly-d-lysine and collagen coated coverslips. Cells were treated with 0.007–3.5 µM recombinant αSYN or 0.1–1.0 µg/ml LPS in growth media for 6 h. After which the cells were fixed in 4 % (w/v) paraformaldehyde in PBS and permeabilized with 1 % (v/v) Triton X-100 for 5 min and blocked in 10 % (v/v) normal goat serum in PBS solution for 60 min at room temperature. Anti-p65 NF-κB (1:100) and anti-mouse-Alexa488 (1:1000) were diluted in 1 % (w/v) BSA in PBS and incubated for 1 h at room temperature or over night at 4 °C in a humidified chamber. Nuclei were counterstained with 2 µg/ml Hoechst 33342 in PBS for 10 min at room temperature. Coverslips were mounted in Mowiol/DABCO solution onto glass slides. Images were acquired with 25× objective with AxioImager microscope equipped with ApoTome Imaging system and processed with AxioVision software. A blinded observer manually counted the number of nuclei positive for NF-κB as well as total nuclei in 200–400 cells per condition.

### Uptake of exogenous αSYN

Primary astrocytes were plated in six-well cell culture plates. The cells were incubated with 0.007–3.5 µM recombinant synuclein, which was added to the growth media. The cells were treated for 1–48 h. In some experiments a 1 h treatment was followed by a recovery time of 0.5–48 h, where the medium was replaced with fresh medium. The cells were washed twice in cold PBS, lysed and processed for Western immunoblot analysis as described above.

### Statistical analyses

Each experiment was performed independently at least three times unless stated otherwise. The statistical analyses were done with JMP 9 or StatView software (both from SAS Institute Inc). Comparisons were analyzed with either unpaired Student’s t test (when comparing differences between genotypes) or two-way ANOVA followed by Fisher’s unprotected least significant difference (PLSD) post hoc test (when assessing inductions after treatments), as stated in the figure legends. P values less than 0.05 were considered as statistically significant.

## Additional files

Additional file 1:
**Figure S1.** NO production in αSYN and TLR4-inhibitor treated astrocytes. Primary astrocytes were treated in OptiMEM (supplemented with penicillin G and streptomycin) with 5 µM of control peptide (ctrl pep; CP7: RNTISGNIYSARRRRRRRRR) or TLR4 inhibitor peptide (viper: KYSFKLILAEYRRRRRRRRR) (both from Imgenex/Biomol) two hours prior to the addition of recombinant αSYN (0.7 µM or 3.5 µM, as indicated) or LPS (0.1 µg/ml) as positive control or left untreated (Ø). 48 h later NO released to the media was measured using Griess reagent (Fluka/Sigma-Aldrich). The amount of NO was normalized to total protein amount in the sample well. The samples were incubated protected from light for 15 min, after which the optic absorption at 550 nm was measured (Model 680 Microplate Reader, Bio-Rad). A standard curve made from 0 to 50 µM sodium nitrite was used as reference. Measurements were normalized against the total protein content of the well. Error bars are standard deviation; n = 16. Additional file 2:
**Figure S2.** Extracellular αSYN induces less p38 MAPK and JNK phosphorylation in *Tlr4* ^−/−^ than in *Tlr4* ^+/+^ astrocytes. Primary astrocyte-rich cultures from littermate *Tlr4* ^−/−^ and *Tlr4* ^+/+^ mice were left untreated (-) or treated with LPS (as positive control) and recombinant human αSYN for one hour, after which medium was replaced and cells let to recover for indicated times **(A)**, or continuously for indicated times **(B)**. Immediately after lysis the cell lysates were immunoblotted for phosphorylated p38 MAPK and JNK. Total p38 MAPK, total JNK and GAPDH serve as controls for equal protein loading. Arrows indicate the heights of JNK2 α2 and β2 (48 kDa, black arrow), JNK1, JNK2α1 and JNK2β1 (44 kDa, grey arrow), and a potentially cross-reacting band possibly from phosphorylated ERK2 (41 kDa, open arrow). Several representative experiments shown for a total number of **(A)** N = 2 (*Tlr4* ^+*/*+^) and N = 9 (*Tlr4* ^−*/*−^) and **(B)** N = 4 (*Tlr4* ^+*/*+^) AND N = 5 (*Tlr4* ^−*/*−^) mice, respectively. Additional file 3:
**Figure S3.** Low levels of contaminating endotoxins and proteins in the recombinant synuclein preparations. (A) Endotoxin levels in the purified protein samples were measured with the 0.06 EU/ml cut off using three dilutions of the samples (1:1, 1:10 and 1:25). Protein concentration was measured with nanodrop using the extinction factors ε_(280, 0.1 %)_ 0.354 for αSYN and 0.417 for βSYN. The table indicates the concentrations of synuclein and endotoxin levels in the protein stocks used in this work. **(B)** Samples of purified recombinant αSYN and βSYN were tested for purity by a sensitive variant of Coomassie stained polyacrylamide gels. **(C)** Samples of purified synuclein proteins were immunoblotted using antibodies specific for αSYN and βSYN, respectively. **(D)** Samples that were cut out from Coomassie stained polyacrylamide gels (indicated by red dashed boxes in the upper right panel) were trypsinized and the peptides were analyzed by mass spectroscopy.

## Abbreviations

COX-2: cyclooxygenase-2
GAPDH: glyceraldehyde-3-phosphate dehydrogenase
IL: interleukin
JNK: c-Jun N-terminal kinase
LPS: lipopolysaccharide
MAPK: mitogen-activated protein kinase
NF-κB: nuclear factor-κB
NO: nitric oxide
NOS: NO synthase
PBS: phosphate-buffered saline
PD: Parkinson’s disease
SYN: synuclein
TLR: toll-like receptor
TNF-α: tumor necrosis factor-α
