# Supplementary figures and images for: Exogenous α-synuclein induces toll-like receptor 4 dependent inflammatory responses in astrocytes
Source: BMC Neurosci. 2015 Sep 7;16:57. doi: 10.1186/s12868-015-0192-0 (PMC4562100; doi:10.1186/s12868-015-0192-0)

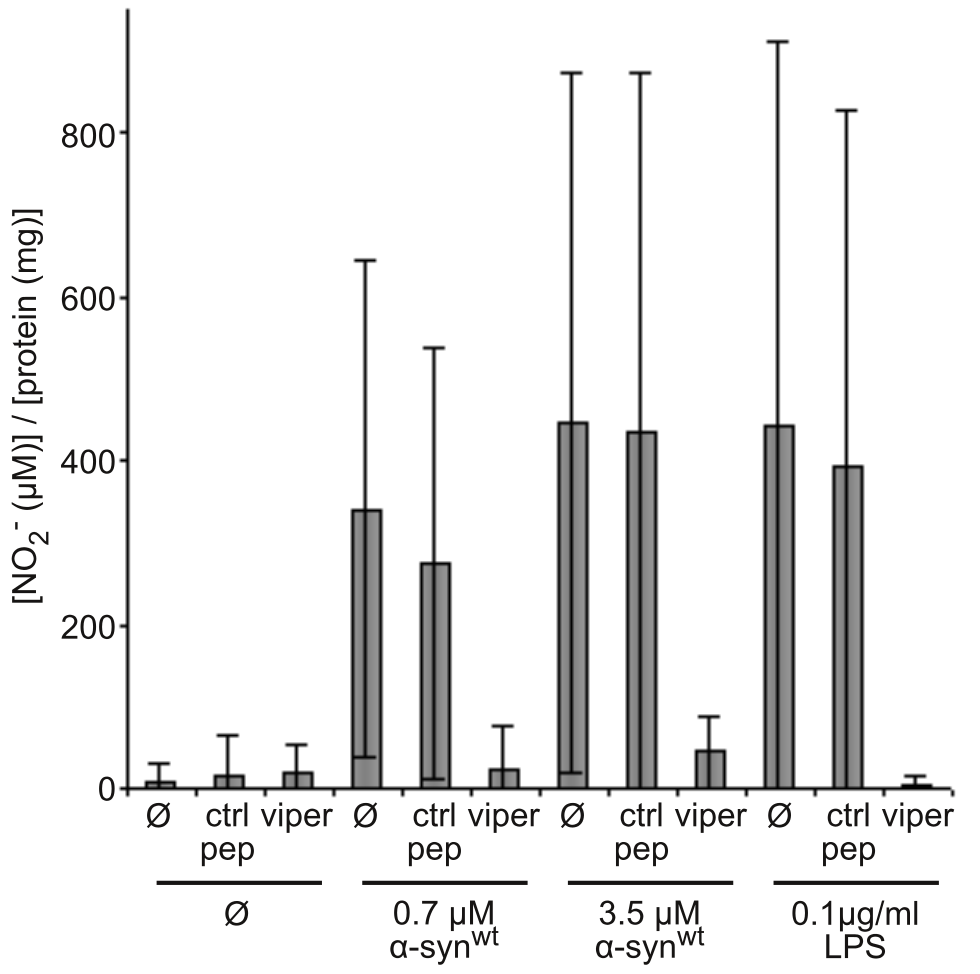

Supplement: Additional file 1: — Figure S1. NO production in αSYN and TLR4-inhibitor treated astrocytes. Primary astrocytes were treated in OptiMEM (supplemented with penicillin G and streptomycin) with 5 µM of control peptide (ctrl pep; CP7: RNTISGNIYSARRRRRRRRR) or TLR4 inhibitor peptide (viper: KYSFKLILAEYRRRRRRRRR) (both from Imgenex/Biomol) two hours prior to the addition of recombinant αSYN (0.7 µM or 3.5 µM, as indicated) or LPS (0.1 µg/ml) as positive control or left untreated (Ø). 48 h later NO released to the media was measured using Griess reagent (Fluka/Sigma-Aldrich). The amount of NO was normalized to total protein amount in the sample well. The samples were incubated protected from light for 15 min, after which the optic absorption at 550 nm was measured (Model 680 Microplate Reader, Bio-Rad). A standard curve made from 0 to 50 µM sodium nitrite was used as reference. Measurements were normalized against the total protein content of the well. Error bars are standard deviation; n = 16. [file 12868_2015_192_MOESM1_ESM.png]

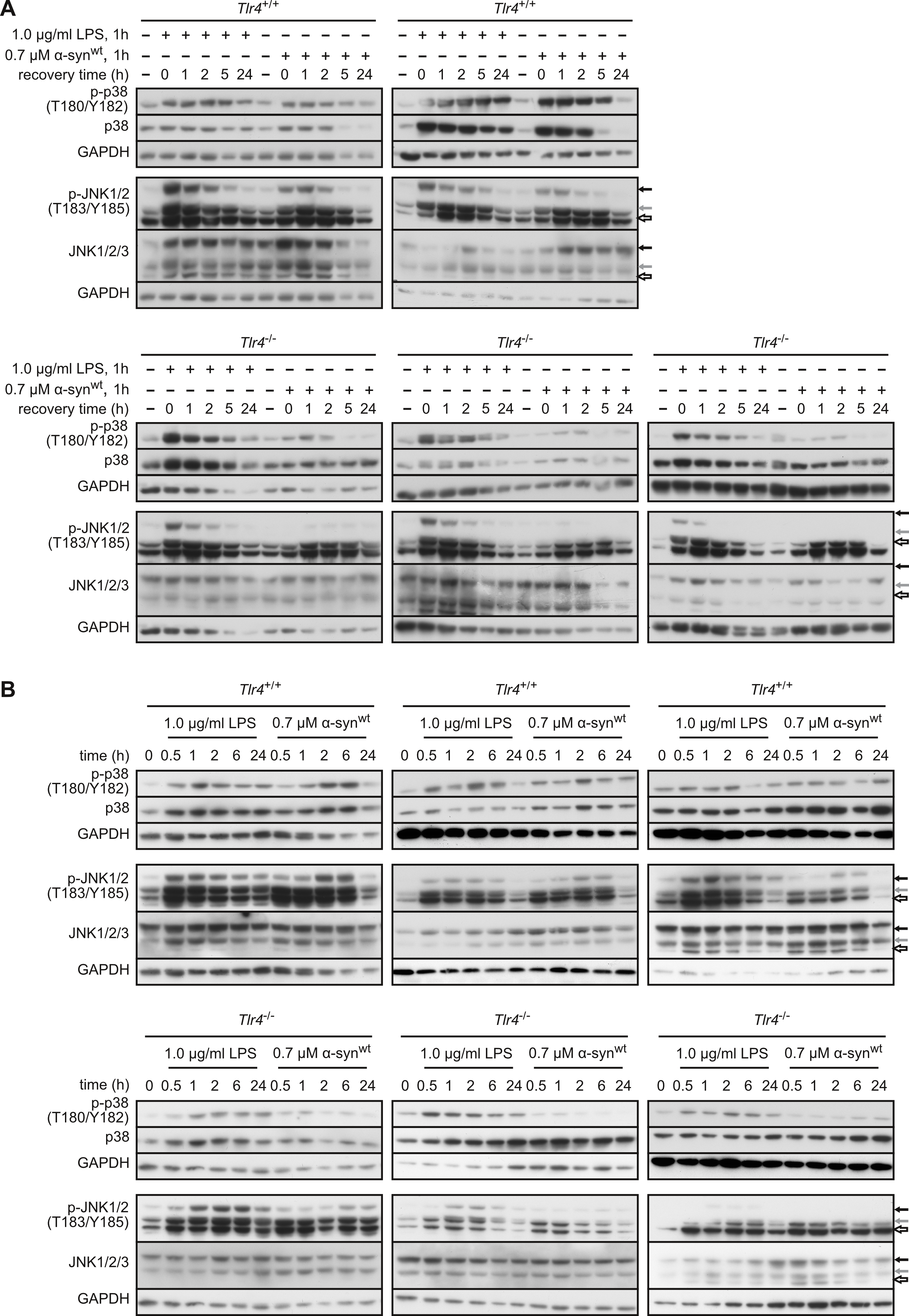

Supplement: Additional file 2: — Figure S2. Extracellular αSYN induces less p38 MAPK and JNK phosphorylation in Tlr4 −/− than in Tlr4 +/+ astrocytes. Primary astrocyte-rich cultures from littermate Tlr4 −/− and Tlr4 +/+ mice were left untreated (-) or treated with LPS (as positive control) and recombinant human αSYN for one hour, after which medium was replaced and cells let to recover for indicated times (A), or continuously for indicated times (B). Immediately after lysis the cell lysates were immunoblotted for phosphorylated p38 MAPK and JNK. Total p38 MAPK, total JNK and GAPDH serve as controls for equal protein loading. Arrows indicate the heights of JNK2 α2 and β2 (48 kDa, black arrow), JNK1, JNK2α1 and JNK2β1 (44 kDa, grey arrow), and a potentially cross-reacting band possibly from phosphorylated ERK2 (41 kDa, open arrow). Several representative experiments shown for a total number of (A) N = 2 (Tlr4 +/+) and N = 9 (Tlr4 −/−) and (B) N = 4 (Tlr4 +/+) AND N = 5 (Tlr4 −/−) mice, respectively. [file 12868_2015_192_MOESM2_ESM.png]

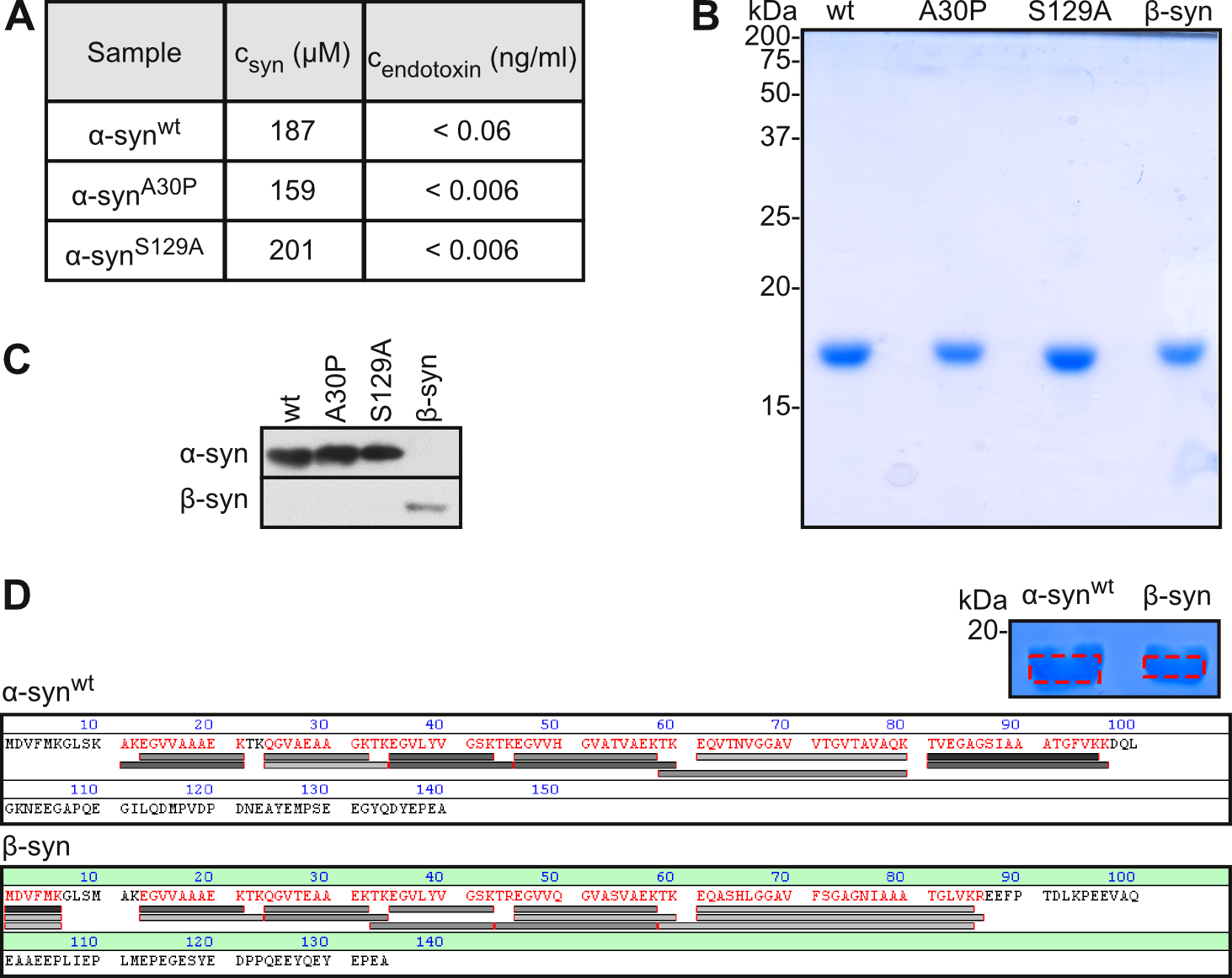

Supplement: Additional file 3: — Figure S3. Low levels of contaminating endotoxins and proteins in the recombinant synuclein preparations. (A) Endotoxin levels in the purified protein samples were measured with the 0.06 EU/ml cut off using three dilutions of the samples (1:1, 1:10 and 1:25). Protein concentration was measured with nanodrop using the extinction factors ε(280, 0.1 %) 0.354 for αSYN and 0.417 for βSYN. The table indicates the concentrations of synuclein and endotoxin levels in the protein stocks used in this work. (B) Samples of purified recombinant αSYN and βSYN were tested for purity by a sensitive variant of Coomassie stained polyacrylamide gels. (C) Samples of purified synuclein proteins were immunoblotted using antibodies specific for αSYN and βSYN, respectively. (D) Samples that were cut out from Coomassie stained polyacrylamide gels (indicated by red dashed boxes in the upper right panel) were trypsinized and the peptides were analyzed by mass spectroscopy. [file 12868_2015_192_MOESM3_ESM.png]
